# Cyberbiosecurity: An Emerging New Discipline to Help Safeguard the Bioeconomy

**DOI:** 10.3389/fbioe.2018.00039

**Published:** 2018-04-05

**Authors:** Randall S. Murch, William K. So, Wallace G. Buchholz, Sanjay Raman, Jean Peccoud

**Affiliations:** ^1^Virginia Tech – National Capital Region, Virginia Polytechnic Institute and State University, Arlington, VA, United States; ^2^Weapons of Mass Destruction Directorate, Federal Bureau of Investigation, Washington, DC, United States; ^3^Biological Process Development Facility, University of Nebraska, Lincoln, NE, United States; ^4^Department of Chemical and Biological Engineering, Colorado State University, Fort Collins, CO, United States

**Keywords:** cyberbiosecurity, bioeconomy, biosecurity, biomanufacturing, cybersecurity, cyber-physical security, supply chain

## Abstract

Cyberbiosecurity is being proposed as a formal new enterprise which encompasses cybersecurity, cyber-physical security and biosecurity as applied to biological and biomedical-based systems. In recent years, an array of important meetings and public discussions, commentaries and publications have occurred that highlight numerous vulnerabilities. While necessary first steps, they do not provide a systematized structure for effectively promoting communication, education and training, elucidation and prioritization for analysis, research, development, test and evaluation and implementation of scientific, technological, standards of practice, policy, or even regulatory or legal considerations for protecting the bioeconomy. Further, experts in biosecurity and cybersecurity are generally not aware of each other's domains, expertise, perspectives, priorities, or where mutually supported opportunities exist for which positive outcomes could result. Creating, promoting and advancing a new discipline can assist with formal, beneficial and continuing engagements. Recent key activities and publications that inform the creation of Cyberbiosecurity are briefly reviewed, as is the expansion of Cyberbiosecurity to include biomanufacturing which is supported by a rigorous analysis of a biomanufacturing facility. Recommendations are provided to initialize Cyberbiosecurity and place it on a trajectory to establish a structured and sustainable discipline, forum and enterprise.

## Introduction

We propose “Cyberbiosecurity” as an emerging hybridized discipline at the interface of cybersecurity, cyber-physical security and biosecurity. Initially, we define this term as “understanding the vulnerabilities to unwanted surveillance, intrusions, and malicious and harmful activities which can occur within or at the interfaces of comingled life and medical sciences, cyber, cyber-physical, supply chain and infrastructure systems, and developing and instituting measures to prevent, protect against, mitigate, investigate and attribute such threats as it pertains to security, competitiveness and resilience.” We emphasize this is an initial definition; we fully expect that the definition and the landscape will rapidly evolve, requiring the definition to be revised. We also contend that, because of its diversity and extent, cyberbiosecurity needs its own systematics, so that it can be better communicated, organized, explored, advanced and implemented. Here, we also posit that cyberbiosecurity contributes to a larger strategic objective of “Safeguarding the Bioeconomy” (The National Academies of Sciences, Engineering and Medicine., 2014), a concept advanced in the U.S., which seeks to increase security and resilience of the Bioeconomy to protect its rapidly changing cyber-life science topology.

Thus, far, what we are proposing to call cyberbiosecurity has primarily been initiated out of two principal sets of activities. The first activities involved a study (American Association for the Advancement of Science et al., 2014) and three workshops (The National Academies of Sciences, Engineering and Medicine., 2014, 2015, 2016) which were primarily focused on security issues with respect to “Big Data” and the relationship with the “Bioeconomy.” The second set was a first-ever systems analysis of a biomanufacturing facility which expands the view to include a different “target set” and approach to understanding vulnerabilities with sharp acuity. This tasked study was conducted to comprehensively understand the vulnerabilities with respect to a wide range of unwanted intrusions and nefarious activities in the life science, cyber, cyber-physical, infrastructure and supply chain aspects, and determine what measures could be taken or developed and implemented to anticipate, detect, identify, prevent, mitigate, respond to and attribute such potential exploitations. The first published paper on cyberbiosecurity primarily focuses on the security of the biotechnology interface with cyberspace (Peccoud et al., 2017). In addition to the system analysis as part of the second set, a small workshop was held in the US that sought to scope and stimulate interest in the government, academic, corporate and non-profit sectors, create a core constituency, understand what topics and themes could constitute cyberbiosecurity, identify priorities and begin to develop a campaign and timeline. The workshop was highly successful. These endeavors, together with additional recent activities and publications (Kozminski and Drubin, 2015; Pauwels and Vidyarthi, 2016, 2017; Pauwels and Dunlap, 2017), have added to scoping the future of cyberbiosecurity yet to come.

## Background

Simply stated, since its inception biosecurity has been primarily focused on reducing the risks associated with the misuse of science which could cause harm to humans, animals, plants and the environment through the creation, production and deliberate or accidental release of infectious disease agents or their byproducts (e.g., toxins). Cybersecurity has been a separate field which has been primarily focused on the security of information technology based systems, from personal computers and communications devices to large infrastructures and networks. Up until just the past few years, the “cyber” overlaps with biosecurity have not been realized or fleshed out. The important interrelationship between biosecurity and cybersecurity is gaining increasing attention. We posit that the two must work collaboratively, and will not be effective working separately. Cyberbiosecurity actually started with thinking about a particular set of problems being confronted by the life sciences. As a result of our recent work, described below, other dimensions are being added. Establishing a unifying discipline, crafting its systematics and identifying an evolutionary path forward are within reach.

The economic strength and growth of the United States have been due to a culture and environment that foster innovation. Those developments could not be possible without significant contributions by science and engineering. The intersection among economic growth and the biological sciences contributions—the bioeconomy—has recently been recognized as an important component of national security. For the U.S., the Bioeconomy accounts for an estimated $4 trillion annually, nearly 25% of the GDP. That contribution ranges from pharmaceuticals to renewable energy, from environmental remediation to public health resilience, and from agriculture to response of emerging diseases. As part of the U.S. national security architecture, “safeguarding the sciences” is a priority. In doing so, the U.S. Federal Bureau of Investigation (FBI) and other Federal Agencies also fulfill the U.S. obligation to the Biological Toxins and Weapons Convention (BTWC) and compliance to the United Nations Security Council Resolution (UNSCR) 1540—preventing the misuse of biological material, technology, and expertise and enforcement of the related statutes. The FBI also sponsors and actively engages the International Genetically Engineered Machine (iGEM) competition to inculcate a culture of security among international students—emerging leaders of research, industry, and policymaking. At the same time the FBI works with U.S. policymakers (You, 2017) to redefine and scope of the biosecurity spectrum for the Twenty first Century—a century where there is unprecedented pace of biological research and innovation, the use of diverse and large datasets—Big Data—to answer global scientific and societal priorities and opportunities. Concomitant to both realized and future benefits and growth, With the life sciences becoming increasingly digitized, and intellectual property protection, cyber intrusion, the protection of personal medical and genomic information, and the impacts on science, trade and commerce loom large. Engagements with the science media (Kozminski and Drubin, 2015) and testimonies (You, 2017) have raised these issues to advance both U.S. competitiveness and national security.

In 2014, the American Association for the Advancement of Science (AAAS), FBI and the United Nations Interregional Crime and Justice Research Institute (UNICRI) published a report entitled “National and Transnational Security Implications of Big Data in the Life Sciences (American Association for the Advancement of Science, et al., 2014). Briefly, this report starts by helping to understand “Big Data”; massive, diverse data sets that are created, reside, are analyzed and move in information ecosystems. For the life sciences Big Data refers to datasets including “raw data, combined data, or published data from the health-care system, pharmaceutical industry, genomics and other –omics fields, clinical research, environment, agriculture, and microbiome efforts.” Further, they state that Big Data also includes analytic technologies and outputs, such as from “data integration, data mining, data fusion, image and speech recognition, natural language processing, machine learning, social media analysis, and Bayesian analysis.” A number of areas that have drawn and need attention are pointed out, such as the security of the cyber infrastructure and data repositories, and the privacy and confidentiality of individuals. In our view, their focus on the security risks of Big Data in the life sciences falling into just two major categories, i.e., inappropriate access to data and analytic technologies through vulnerabilities in the data and cyber infrastructure; and, the use of Big Data technologies to integrate current data and enable the design of a harmful biological agent should be revisited and refined. Thanks to this team's efforts, not only do we have a useful topology of Big Data, the beginnings of a structure for thinking about security implications at the bio-cyber interface (Technical, Legal, Institutional and Individual) and a set of high-level recommendations for a path forward.

From 2014 to 2016, three workshops were organized by the U.S. National Academies on behalf of the FBI under the theme of “Safeguarding the Bioeconomy.” The first (The National Academies of Sciences, Engineering and Medicine., 2014) laid the foundation for the next two. Presentations and discussions focused on the security implications of the convergence in the life and chemical sciences with physical, mathematical, computational, engineering, and social and behavioral sciences. In addition to broader contexts, two specific technologies received focus: neuromorphic computing and 3-D bioprinting. The second workshop (The National Academies of Sciences, Engineering and Medicine., 2015) introduced a range of new threats to and vulnerabilities of the Bioeconomy which at the time had not received focused consideration with respect to U.S. “competiveness, security, economic growth and global leadership in research and innovation.” This workshop was built on three major themes: The Role of Informatics in the Bioeconomy, Criminal Threats and Vulnerabilities in the Existing and Near-Future Bioeconomy and Securing and Flourishing the Bioeconomy for the Future. Rapid growth of this sector creates increasing security risks to proprietary materials and informatics, industrial espionage and data hacks are increasing in frequency, and traditional security measures are increasingly ineffective. Still, alternative and adaptive security measures could be implemented even with the inherent openness of emerging technologies upon which the Bioeconomy is dependent. Workshop participants not only provided more detail on the threats and vulnerabilities but also both comprehensive categories and specific approaches that could be taken to address the problems and concerns identified. The third workshop (The National Academies of Sciences, Engineering and Medicine., 2016) principally focused on data generation and access with respect to the Bioeconomy within several categories of both clinical and non-clinical data, from the perspectives of Biosecurity, Data Policy and Regulation, Future Implications, Technology Advances, Data Sovereignty and Sharing, Cybersecurity and International Implications. Taken together, these events significantly expanded the view of what the emerging discipline of cyberbiosecurity could encompass.

Pauwels and her co-authors also raise important concerns and recommendations for the security of biotechnology in cyberspace. In the first, she and Vidyarthi (Pauwels and Vidyarthi, 2016) raise concerns over data breaches of health care information and what it means for the biotechnology industry. Protecting digital DNA and personal medical information is highlighted and the fact that a then recent U.S. Presidential cybersecurity initiative put significant resources into shoring up cyberinfrastructure. Unfortunately, the need for improvements to protecting the Bioeconomy, which is heavily dependent on information systems and infrastructure, was not recognized. The report outlined the implications of not protecting the Bioeconomy dimension. Their recommendations were primarily focused on protecting genomic data. In the second report, Pauwels and Dunlap (2017) go into more depth framing potential cyber-vulnerabilities for specific types of biotechnologies: genome-editing; DNA assembly, synthesis and printing; portable genomic sequencers; artificial intelligence for understanding biological complexity; autonomous systems and robotics in cloud labs; and, lab-on-a-chip and microfluidic technologies all of which have cyber-physical interfaces. These authors also suggest governance systems and policy recommendations which might be harnessed to address the lab-focused concerns they raise.

Other recent publications also highlight the complexity of the enterprise we are terming “Cyberbiosecurity” and concerns over security, robustness and resiliency. These include security of personal genomic data when foreign companies that have purchased all or part of U.S. companies or have been contracted for genomic or health care data services which provides access to sensitive personal information (Pauwels and Vidyarthi, 2017), the continuing vulnerability of electronic health records (Weise, 2015) and health care systems (Hackett, 2015; Winton, 2016; Griffin, 2017), imposing control over DNA sequencing through DNA-encoded malware (Greenberg, 2017), synthetic biology supply chain vulnerabilities (Frazer et al., 2017), cyber compromise of large industrial biopharma (Collier, 2017; Shaban and Nakashima, 2017), and high-level studies which are systematically examining U.S. biodefense programs and capabilities (The Blue Ribbon Study Panel on Biodefense, 2015; Center for the Study of Weapons of Mass Destruction, 2017). The Dark Web/Dark Net (Beckett, 2009; Interpol, 2015; Langewiesche, 2016) could be included as it interfaces with dual use life science endeavors and biopharma research, development, intellectual property and products and compromise of the integrity of critical life science and health cyber-supported technologies and infrastructures. Because of the reliance on bioinformatics, the security of synthetic DNA could also be included, as well (Adam et al., 2011). Clearly, this rapidly expanding galaxy does needs a universally accepted definition, common terms of reference, and defined boundaries and structure for best value, ordered evolution and impact.

## Adding another dimension: cyberbiosecurity systems analysis of a biomanufacturing facility

Now we add another dimension to cyberbiosecurity, and take an approach that we posit that should be incorporated with other aspects discussed earlier. The biopharma industry itself has its own substantial equities and investments in the research, development, production and sale of vaccines, therapeutics and prophylactics for the global market. The U.S. Government has substantial investments in the development and production of critical vaccines and biotherapeutics for both civilian and military purposes. Concomitantly, experts are increasingly recognizing that biomanufacturing itself is potentially vulnerable to unwanted or illicit activities which could result in damaging outcomes. These could include the theft of intellectual property, disruption of the supply chain, manipulation of the bioprocess development and bioproduction, cyberattacks on key information technology components and cyberphysical interfaces, the corruption of critical data and manipulation of security systems and infrastructure upon which secure and safe facility operations are dependent. Our sponsor was not interested in generalizations or esoteric approximations about the security vulnerabilities of a biomanufacturing facility but wanted a comprehensive, detailed, actionable analysis.

Thus, we undertook an in-depth, multidimensional analysis of an existing biomanufacturing facility to identify security gaps and vulnerabilities, make recommendations with respect to addressing those identified and projected and set the stage for more specific and comprehensive measures to be undertaken, whether they exist or have to be developed and validated. The systems analysis approach used was designed to assess the state of security at present, determine what an acceptable state of security would be, and provide guidance and recommendations to take the facility from its current state to the desired state.

The bioprocess development/bioproduction facility used as the “test bed” for this analysis designs, develops and produces clinical trial quantities of protein-based biotherapeutics and the associated documentation for commercial and government clients. If the outputs from this “test bed” meet client expectations and the client receives government approval, the client scales up production and the product is marketed. This facility was studied as a system, consisting of four key, interrelated subsystems: end-to-end bioprocess development/biomanufacturing; the supply chain; the supporting information systems infrastructure and cyber-physical interfaces with bioprocess development and biomanufacturing; and, facility infrastructure including its relationship to the facility's host infrastructure. The systems analysis was a phased process with project management methods applied. The facility or any of its components or operations were not compromised, corrupted or altered during this project in any manner or form. Rather, it was studied thoroughly yet benignly. The analysis included human factors and “downstream” considerations, as well.

**Figure 1:**
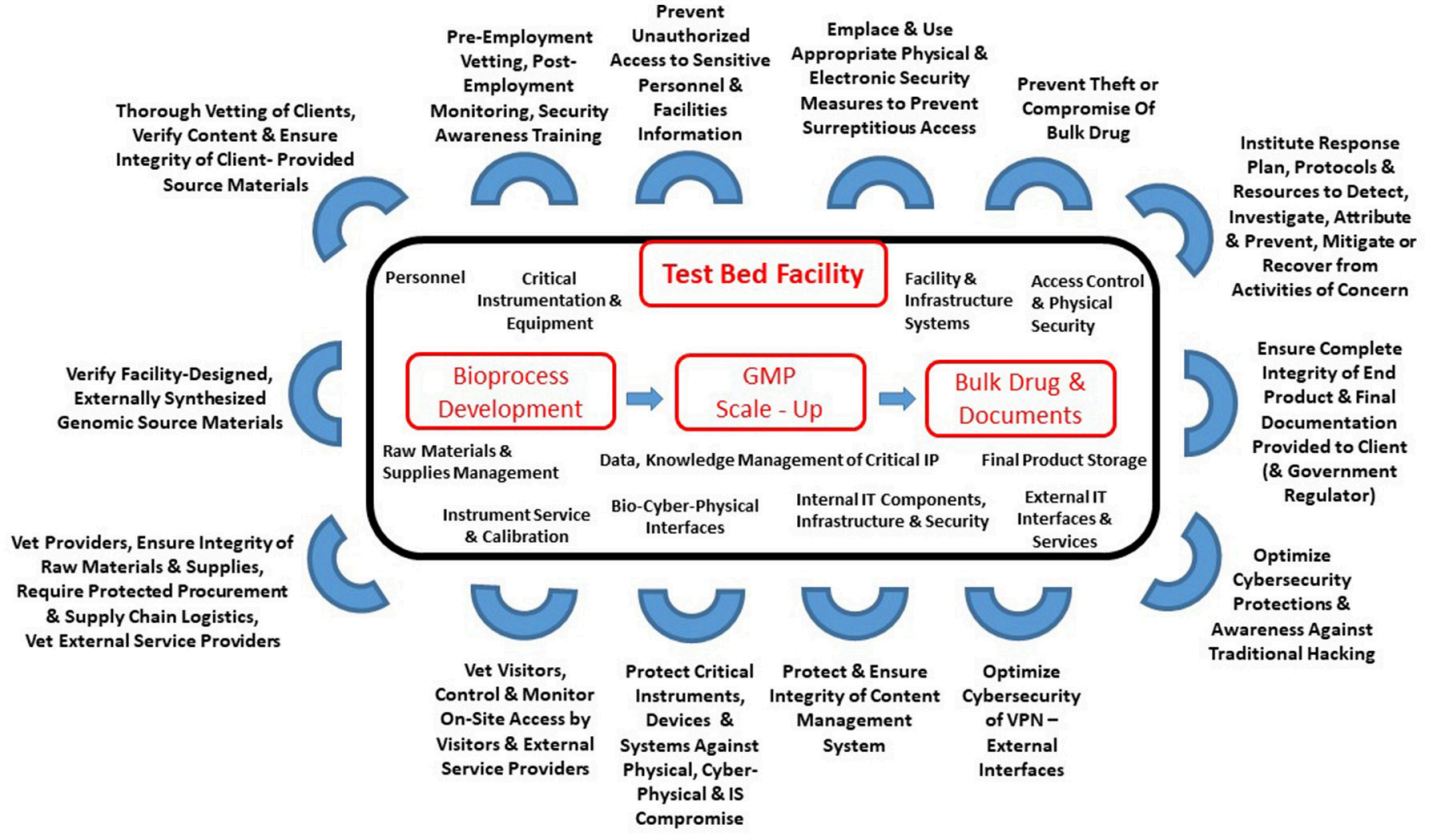
A systems view of protecting a biomanufacturing facility. For each defensive set identified, multiple threats and impacts were identified and potentially more than one pathway or technique could be used by an adversary to achieve their objectives. GMP, Good Manufacturing Practice; IT, Information Technology; IS, Information Systems; VPN, Virtual Private Network; IP, Intellectual Property.

The systems aspects of a biomanufacturing facility which are potentially vulnerable to security threats and the solutions required are summarized in Figure 1.

Due to space limitations, we provide only a top-level view of the analysis. Key overarching findings include:

- Vulnerabilities can exist across the entire system, from bioprocess development and GMP to supply chain, to cyber-physical and infrastructure; there are potentially more than one might anticipate *a priori*.
- Successful exploitation of vulnerabilities can occur through passive and active means for passive and active purposes, depending upon adversaries' intentions, objectives, accesses, knowledge and resources, and outcomes sought.
- Exploitation of some vulnerabilities require direct access to facilities or components; personnel and physical security aspects should not be overlooked.
- Adversaries can use combinations and sequences of methods and targeting, both subtle and not, to attempt to and achieve their objectives.
- The operational capabilities of adversaries, not just technical, must be considered and accounted for in planning for and implementing security measures.

We emphasize that, while there are general principles that apply and observations that will derive from such analyses, the analysis design and execution and the resulting solution set, should be tailored on a facility-by-facility basis. We note that the defensive areas noted may not be singular, but require combinations of defensive approaches and techniques to be identified and implemented to ensure optimal security robustness.

What is considerably important from this analysis is that a rigorous study of a facility such as this can result in the identification and characterization of discrete vulnerabilities, gaps, shortfalls and opportunities for which readily-available solutions can be implemented, or otherwise can be developed, tested and implemented. We did not conduct detailed studies regarding how genomics can be compromised as it relates to biomanufacturing, because we were directed not to but are well aware of plausible scenarios and what the effects could be.

Our analysis demonstrates that biomanufacturing facilities can benefit from comprehensive, multidisciplinary analyses to identify security vulnerabilities leading to solutions to mitigate or address them. This, in turn, raises the prospect of the development and validation of a set of methods or protocols would be in order which could be used by facility staff or external service providers to shore up individual facilities from Do-It-Yourself to large Biopharma. Walking this out, guidelines or standards could be developed, established and accepted to ensure consistency and quality of the analyses conducted, the credentials of the personnel doing so and the quality and effectiveness of measures undertaken. While sophisticated adversaries could design and execute sophisticated attacks, it is likely in many instances that relatively straightforward methods and practices could raise the bar considerably to reduce risk. Lastly, combining analyses of this sort could be used as a basis for informed investments in research, development, test and evaluation for solutions to the most worrisome current and future threats.

## Moving cyberbiosecurity forward

Many other critical cyber-enabled life science and biomedical technologies, systems and applications naturally lend themselves to inclusion within Cyberbiosecurity. These include, but are not limited to, personalized genomics, and medical and fitness technologies, 3-D printing of critical personalized medical devices, and medical laboratory and surgical robotics. A more comprehensive system is warranted. Cyberbiosecurity could be expanded to include cyber-bio systems within agriculture and farm-to-table food production, processing and distribution systems, and within natural resource and environmental management. Direct and ordered engagements of the pertinent sectors of the life sciences - biosecurity and cyber-cybersecurity communities, should occur. Academia, industry, government or non-profits (including policy, regulatory and legal experts) need to begin to learn to communicate with and educate each other, harmoniously identify and develop priorities, opportunities and specify “next steps.” A major opportunity exists right now to propose a unified structure and common vernacular. Lastly, while definition and assemblage of Cyberbiosecurity is occurring, national or international strategies should be pursued to harmonize the emerging enterprise and foster measurable value, success and sustainability.

## Author contributions

RM: Co-originator of cyberbiosecurity concept; Lead author, responsible for structure, content and figure; responsible for considering, incorporating co-author contributions and suggested modifications; responsible for final version. WS: Co-originator of Safeguarding the Bioeconomy concept and campaign; contributed or reviewed and edited paragraphs related to the AAAS-FBI-UNICRI Big Data report, the US National Academies workshops and the relevant FBI programs and initiatives. SR: Co-originator of cyberbiosecurity concept; overall quality assurance and readability reviews and modifications; contributions to section on cyberbiosecurity applied to biomanufacturing. WB: Contributions to section on cyberbiosecurity applied to biomanufacturing; reviews to ensure content quality and readability of paper. JP: Co-originator of cyberbiosecurity concept; review and critique of manuscript to ensure complementarity and alignment with first paper published on cyberbiosecurity (biotechnology focus) in another journal (he is the lead author; RM, SR, and WB are co-authors).

### Conflict of interest statement

The authors declare that the research was conducted in the absence of any commercial or financial relationships that could be construed as a potential conflict of interest.
